# Revisiting Cardiac Biology in the Era of Single Cell and Spatial Omics

**DOI:** 10.1161/CIRCRESAHA.124.323672

**Published:** 2024-06-07

**Authors:** Jack A. Palmer, Nadia Rosenthal, Sarah A. Teichmann, Monika Litvinukova

**Affiliations:** Wellcome Sanger Institute, Wellcome Genome Campus, Hinxton, Cambridge, United Kingdom (J.A.P., S.A.T.).; Wellcome-MRC Cambridge Stem Cell Institute, Jeffrey Cheah Biomedical Centre, Cambridge Biomedical Campus (J.A.P., S.A.T.), University of Cambridge, United Kingdom.; Theory of Condensed Matter Group, Department of Physics, Cavendish Laboratory (S.A.T.), University of Cambridge, United Kingdom.; The Jackson Laboratory for Mammalian Genetics, Bar Harbor, ME (N.R.).; National Heart and Lung Institute, Imperial College London, United Kingdom (N.R.).; University Hospital Würzburg, Germany (M.L.).; Würzburg Institute of Systems Immunology, Max Planck Research Group at the Julius-Maximilians-Universität Würzburg, Germany (M.L.).; Helmholtz Pioneer Campus, Helmholtz Munich, Germany (M.L.).

**Keywords:** cardiovascular diseases, genomics, heart, human, single-cell analysis, spatial analysis

## Abstract

Throughout our lifetime, each beat of the heart requires the coordinated action of multiple cardiac cell types. Understanding cardiac cell biology, its intricate microenvironments, and the mechanisms that govern their function in health and disease are crucial to designing novel therapeutical and behavioral interventions. Recent advances in single-cell and spatial omics technologies have significantly propelled this understanding, offering novel insights into the cellular diversity and function and the complex interactions of cardiac tissue. This review provides a comprehensive overview of the cellular landscape of the heart, bridging the gap between suspension-based and emerging in situ approaches, focusing on the experimental and computational challenges, comparative analyses of mouse and human cardiac systems, and the rising contextualization of cardiac cells within their niches. As we explore the heart at this unprecedented resolution, integrating insights from both mouse and human studies will pave the way for novel diagnostic tools and therapeutic interventions, ultimately improving outcomes for patients with cardiovascular diseases.

The adult human heart comprises a heterogeneous population of approximately 5 billion cells that function in unison in specialized tissue microenvironments (niches) to coordinate each heartbeat. This remarkable feat of orchestration is compromised when cardiac cell function is perturbed by high blood pressure, blood clots, heart attacks, and other comorbidities of cardiovascular disease, which are the leading cause of mortality worldwide.^1,2^ The clinical challenges to effective replacement and regeneration of cardiac tissue stem largely from our insufficient knowledge of the distinct cardiac cell identities, the specific roles they play in normal cardiac function, and how these functions are disrupted in disease.

A diverse range of techniques have been used to explore tissues, including histology, immunofluorescence, and medical imaging. At the cellular level, bulk assays are used to investigate a range of properties, including electrophysiology, metabolism, and biophysics. However, these studies are limited to specific cell types, proteins, or genes, restricting the exploration of cardiac tissue microenvironments.

The recent rise of single-cell technologies has offered the opportunity to take a broader perspective and explore cardiac cells at scale, considering a vast array of cell types and cellular heterogeneity. New spatial transcriptomic technologies offer the opportunity to study cells in molecular detail in situ. Harnessing the power of cross-species studies in mouse and human, these techniques have added increasing molecular detail to tissue: resolving new cell types, building whole-organ reference atlases to facilitate drug target prediction, and elucidating the crucial nature of tissue niches.

Here, we present 3 intersecting views of how the application of these new technologies to cardiac research is transforming the field: Approach, in which we discuss specific experimental and computational advances and best practices when applied to cardiac tissue; System, in which we review comparative analyses of human and mouse heart structure-function relationships and the potential implications for clinical interpretation; and Context, in which we explore the cardiac cells and the microenvironments they create (Figure 1). Integrating these areas of study highlights how recent progress in the field is providing an increasingly comprehensive view of the cellular landscape of the heart.

**Figure 1.**
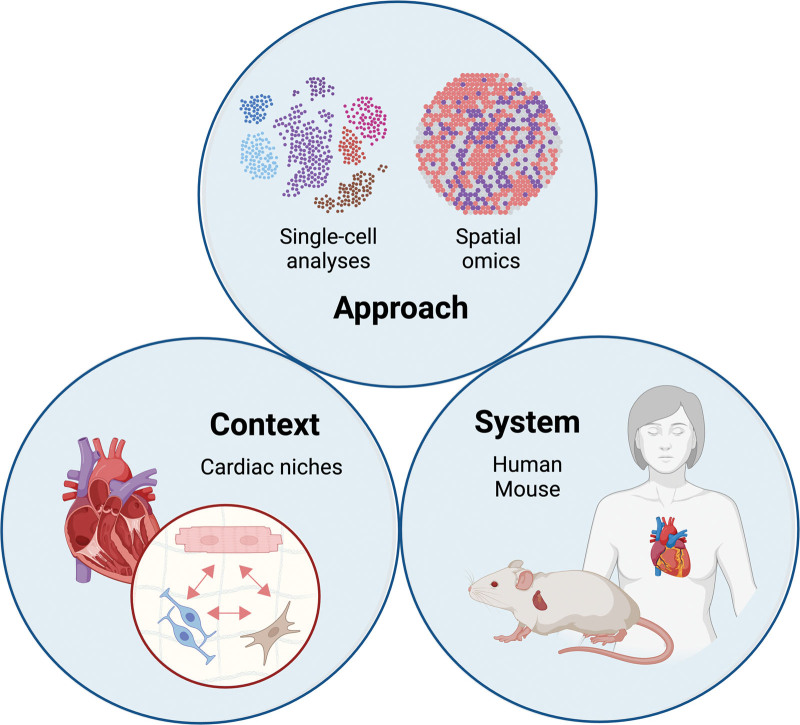
The combination of single-cell and spatial omics technologies in human and mouse cardiac systems to reveal the role of tissue niches in cardiac cell biology.

## APPROACH: EXPLORING CARDIAC CELLS WITH SINGLE-CELL AND SPATIAL TRANSCRIPTOMICS

The rise of single-cell omics has provided the tools to delve deeper into cellular identity and function. Commonly used commercial single-cell multiomic assays allow us to understand not only patterns of gene expression in each cell (scRNA-seq, single-cell RNA sequencing), but also chromatin accessibility (ATACseq, transposase- accessible chromatin with sequencing) and surface protein expression (CITE-seq, cellular indexing of transcriptomes and epitopes).^3^ Other assays, such as single-cell DNA sequencing and chromatin configuration, provide insights into the nuclear spatial context. Integration of these modalities using computational methods can offer deep insights into processes such as regulation of gene expression and genome architecture.

**Figure 2.**
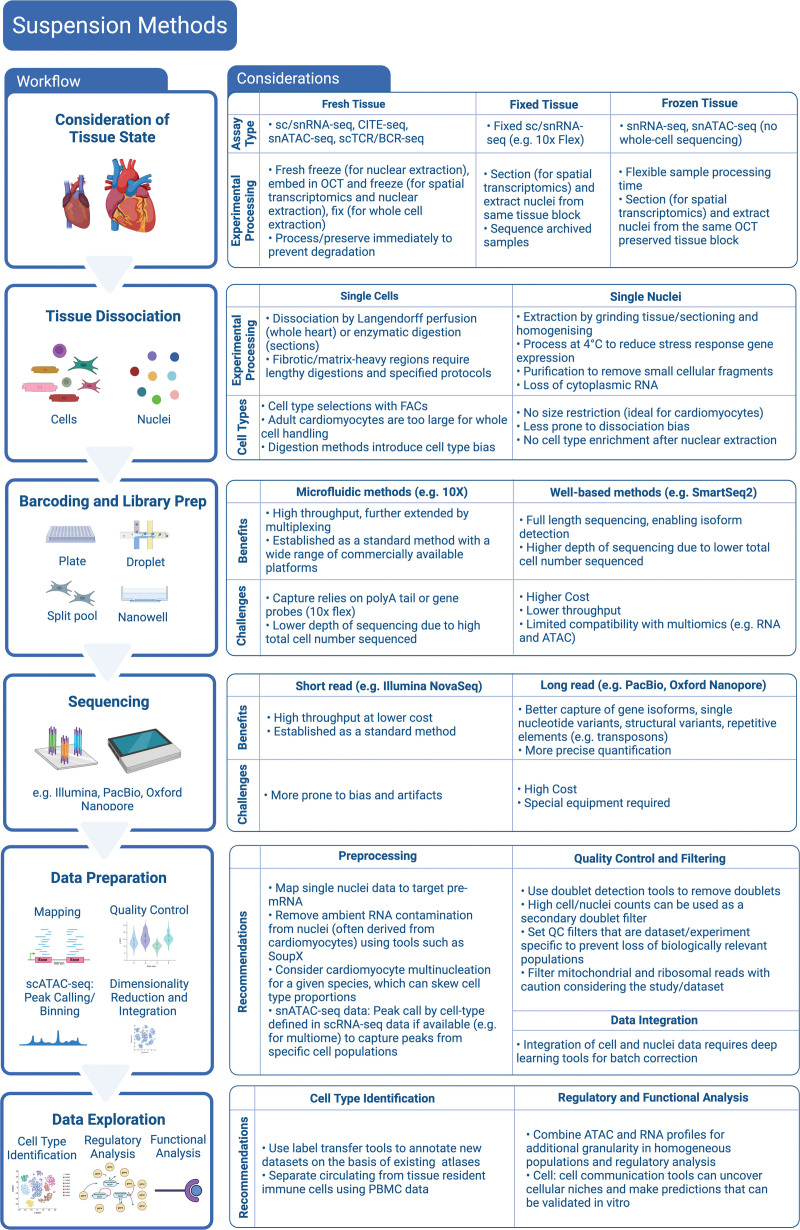
**Workflow of suspension omics methods and considerations and recommendations for each of the steps.** CITE-seq indicates cellular indexing of transcriptomes and epitopes; FAC, fluorescence-activated cell; OCT, optimal cutting temperature; PBMC, peripheral blood mononuclear cells; scRNA-seq, single-cell RNA sequencing; scTCR/BCR-seq, single-cell T-cell receptor/B-cell receptor sequencing; snATAC-seq, single-cell transposase-accessible chromatin sequencing; and snRNA-seq, single nucleus RNA sequencing.

**Figure 3.**
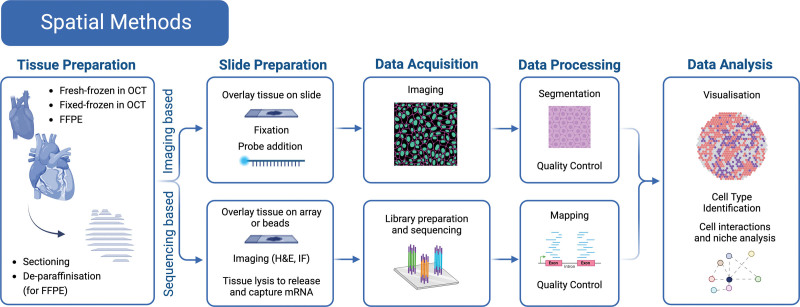
**Overview of the spatial methods and an experimental and computational workflow.** FFPE indicates formalin fixed paraffin embedded; H&F, hematoxylin and eosin; IF, immunofluorescence; and OCT, optimal cutting temperature.

Suspension methods involve on dissociating cardiac tissue to single cells or nuclei and have been the foundation for studying cardiac cells in an isolated context, ranging from time-resolved studies of mouse myocardial infarction (MI)^4–6^ and early stages of heart development^7^ to large-scale atlasing of the human heart,^8,9^ where major cardiac cell compartments and cell states are analyzed at scale. However powerful the single-cell approach, the process of tissue dissociation removes cells from their spatial context, which eliminates a rich source of functional and niche information. Spatial-omic methods provide an assessment of distributions of gene expression within tissue but lack single-cell resolution or breadth of gene profiling that suspension methods offer. Figures 2 and 3 outline the experimental and computational workflows for suspension and spatial methods.

The richest data sets of cardiac cells to date combine multiple modalities to achieve single-cell depth with spatial resolution^10,11^ by assembling a detailed atlas of cell states in suspension and placing them in spatial context to uncover previously unknown microenvironments. Below, we summarize the major challenges as well as the considerations and implications of both suspension and spatial omics in cardiac tissue.

### Challenges in Cardiac Single-Cell Analysis

#### Experimental Challenges

While the varying requirements for isolation and maintenance of the heterogeneous range of cell types found in every tissue introduce an intrinsic bias into any single-cell suspension protocol, the heart presents unique challenges. Postmitotic cardiomyocytes constitute only a third of the cells in the adult mammalian heart, yet they are the predominant cardiac cell type due to their large size, which presents challenges for single-cell isolation.^12^

Cardiac cells have been extensively studied using scRNA-seq analysis,^13–16^ however cell isolation techniques impose size constraints, particularly in popular droplet-based sequencing methods that exclude large cell types, such as cardiomyocytes. Some nanowell approaches, such as ICell8,^17^ have successfully captured intact cardiomyocytes as an alternative targeted approach with limited throughput.^18^ Consideration should be taken to choose an appropriate dissociation strategy that aligns with experimental aims: digestions should always be adjusted for a specific tissue, region, and cell type of interest, and different enzyme mixtures and approaches can preferentially yield different cell types.

Integrating different modalities, scRNA-seq can be combined with antibody labeling of single cardiac cells, either as a selection step or combined with scRNA-seq, such as in CITE-seq, which has been successfully applied to nonmyocyte fractions such as fibroblasts, vascular, and immune cells.^19^

Although cytoplasmic RNA analysis provides the most accurate assessment of protein-coding gene expression, nuclear extraction enables snRNA-seq (single-nuclear RNA sequencing) of larger cells, such as cardiomyocytes.^20,21^ Since the nuclear envelope protects transcripts, fixed or frozen samples can be used, including archived samples, dramatically increasing the pool of available samples.^22^ In addition, nuclei can be used for chromatin accessibility assays such as snATAC-seq (single nucleus transposase-accessible chromatin with sequencing) can be performed on the single-cell level.

Following the creation of a suspension, a library of reads for each cell/nucleus is produced, during which a barcode is added that uniquely identifies reads to a given cell/nucleus of origin. Whole cells can be sorted into individual wells by fluorescence-activated cell sorter, with ligation of unique barcodes and addition of polymerase chain reaction primers in each well (eg, Smart-seq3^23^). This technique yields whole RNA molecules but is limited in cell number due to constraints of plate size. However, the smaller number of cells can be later sequenced to a higher depth. In contrast, droplet-based methods use fluidic systems to encapsulate individual cells in an oil droplet with a gel bead that captures reads using oligo-adaptors.^24,25^ This approach sequences greater cell numbers compared with plate-based methods, but at a shallower sequencing depth per cell, with exclusively 3′ or 5′ reads. The desire to profile many cells across various cell types makes droplet methods a popular approach in cardiac cell analysis, especially for atlasing projects.

Libraries are sequenced using next-generation sequencing technologies which vary in cost, throughput, and accuracy. Sequencing by synthesis (eg, Illumina platforms^26^) has been the most common approach for the cardiac cell studies reviewed here. Long-read sequencing using Oxford Nanopore^27^ or PacBio^28^ offers the potential for transcript isoform as well as parent gene calling, which can be attractive for many research questions. Until recently, this has been technically challenging and prohibitively expensive. With reducing flow cell costs and the release of commercial kits to generate compatible libraries from 10× cDNA^29^ long-read sequencing is gaining in popularity.

#### Computational Challenges

The rise of single-cell technologies has led to the development of specialized tools to analyze the large data sets produced. Recent reviews have addressed the current best practices for analyzing single-cell transcriptomic^30^ and multiomic^31^ data. Here, we briefly outline the major considerations for cardiac single-cell data analysis.

Ambient RNA is caused by the free-floating RNA molecules from lysed cells and can be particularly relevant in single-nuclei experiments.^32^ While purification and washing methods might alleviate ambient RNA to a certain extent, highly abundant cardiac transcripts, such as titin, light and heavy myosin chains, and natriuretic peptides, may still appear in the nonmyocyte compartments. Therefore, computational tools are required to correct for ambient RNA contamination, such as SoupX and CellBender.^33,34^

Droplet-based technologies are particularly sensitive to multiplet formation: the encapsulation of two or more cells within the same droplet. Incomplete tissue dissociation can exacerbate this effect, particularly for the cell pairs that form close connections.^35^ Doublet detection methods that model multiplet profiles as a combination of major cell type signatures^36^ are helpful for detecting this. However, manual annotation is still required, particularly to distinguish doublets from transitional cell states.

Cardiac data sets often require integration of data from multiple samples (time points^37^ or heart regions^9,10^), technologies (scRNA-seq combined with snRNA-seq^9,10^) or modalities (snRNA-seq combined with snATAC-seq^10,11^). Integration into a unified representation is required to ensure a fair representation of as many cell types from a given tissue as possible. These probabilistic (ie, BBKNN^38^) and deep learning (ie, scVI^39^) based methods integrate the data, removing technical variation and retaining biological information. It is important to consider the type of data and the biological task at hand when evaluating the results of these methods. In general, deep learning methods have shown better results when integrating and annotating data from multiple technologies while still preserving the biological information, such as regions or time points.^40^

Translating the findings from one species to another can provide additional challenges, including the correct gene homology and the correct integration of different species. The major technologies for reliable integration of species have been benchmarked recently by Song et al.^41^ New tools, such as SATURN,^42^ integrate the protein space to bypass the need for gene homology, allowing for the integration of more evolutionary distant species while providing species-specific cell types.

Single-cell data sets of cardiac cells are leveraged in a variety of applications. Cardiac atlases that characterize cellular subpopulations in depth can be used to annotate previously identified cell types in new data sets using label transfer, supplemented by the manual annotation of new and transitional states.^43,44^ To account for the identification of marker genes being influenced by the method of isolation (cells or nuclei), statistical models considering those covariates must be used. Furthermore, these data sets can be used to guide further study of cardiac cells by identifying putative drug targets, gene regulatory networks of cardiac transcription factors, and predicting their binding motifs.^10,11^ However, it is important to note that the predictions or inferences made by these sophisticated analyses require validation, such as in vitro functional assays or testing gene expression at the protein level by immunofluorescence.

### Expanding Opportunities in Spatial Tissue Analysis

A comprehensive understanding of cardiac function and disease requires an analysis of distinct cardiac anatomic regions and their interconnections. Histology and fluorescence microscopy are common approaches to evaluate myocardial composition, structure, and pathophysiological changes. However, the low plexity of these techniques restricts exploration to a limited number of proteins or RNA molecules. Spatial-omic methods offer the opportunity to map the whole transcriptome within the tissue context, assigning vast and detailed gene expression patterns to cardiac structures, exploring the localization of specific cell types and their gene expression, potential molecular mechanisms for cell-cell communication, and even regions of specific signaling.

Spatial transcriptomic methods have been used to analyze key human cardiac structures in development^45^ alongside tissues from the epicardium and conduction system^10^ to distinct zones of cardiac infarcts.^11^ These studies use sequencing-based spatial methods where tissue sections are placed over a slide with microarray spots containing clustered oligonucleotides with positional barcodes. Tissue is lysed over the array to release RNA molecules that bind to oligos within spots. An alternative approach is Slide-seq, where tissue is lysed on an array of beads barcoded with positional information.^46^ Both methods require hematoxylin and eosin staining of tissue samples to contextualize spot location.

Commercially available sequencing-based spatial methods, such as 10x Visium, do not achieve true single-cell resolution yet. Probe-based spatial methods rely on imaging and can resolve single cells, contingent on microscope resolution. Commercial platforms such as Vizgen MERSCOPE (based on MERFISH^47^) and 10× Xenium (based on rolling circle amplification^48^) overcome the limits of fluorophore multiplexing by using barcoded probes that are decoded via successive rounds of imaging. Although the plexity of these techniques has dramatically increased in recent years, the number of genes that can be detected is limited by probe availability and cost. These platforms offer future opportunities for spatial transcriptomics of cardiac tissue at single-cell resolution.

The increasing application of spatial transcriptomic methods to tissue has demanded suitable computational analysis tools to handle the data generated. Best practices are being defined,^49,50^ but they are likely to evolve in line with increased data generation by these methods. The use of single-cell atlases to deconvolve the cell types captured at the individual spots is an essential data analysis tool used in cardiac tissue studies. Tools such as Cell2Location^51^ and SquidPy^50^ provide analytical suites that allow clustering and statistical testing for cell colocalization and the identification of microenvironments.

Cardiac tissue presents several challenges that should be considered when applying spatial transcriptomic methods. The myocardium can appear relatively homogeneous to the eye, and the identification of the area of interest can be challenging, particularly when anatomic hallmarks are not available. It is always advisable to first use hematoxylin and eosin staining and histological annotation when focusing on a specific area, such as the sinoatrial or atrioventricular nodes. Myocardial tissue is also known to show prominent autofluorescence due to its high metabolic activity and the accumulation of lipofuscin,^52^ a nondegradable protein present in postmitotic cells. This is likely to affect the signal direction in the probe-based fluorescent approaches. Tissue clearing approaches^53^ may alleviate this issue, but risk interference with spatial transcriptomic chemistry.

Considerations of gene plexity, resolution, and throughput must be weighed against the cost of technique, tissue state, and biological questions. We refer the reader to the comprehensive review by Kiessling and Kuppe^54^ for an evaluation of existing techniques.

## SYSTEMS: COMPARING MOUSE AND HUMAN CARDIAC CELLS

Studying human cardiac tissue directly provides a more accurate representation of human physiology and pathology and is essential for uncovering species-specific aspects of cardiobiology and understanding diseases in a clinically relevant context. However, accessing human samples poses challenges due to ethical considerations, limited availability, and the potential impact of comorbidities.

Animal model systems provide access to healthy and diseased cardiac tissues. The laboratory mouse is widely considered the model organism of choice for studying cardiac structure-function relationships in humans, with whom they share 99% of their genes as well as most physiological and pathological features. Comparative analyses have provided insight into common gene regulatory circuitry across mammalian species,^55^ and gene classifications developed through human and mouse essentiality screens serve as a resource for disease gene discovery.^56^ These advances have guided powerful genomic manipulations in the mouse to generate multiple genetic models of human cardiac pathologies.

Murine hearts share major characteristics with their human counterparts, including the anatomy of the four-chamber structure, similar embryonic development, cell lineages, and major cellular compartments. However, the murine heart develops much more rapidly and beats significantly faster, and its size, structure, and metabolic processes differ from the human heart. These distinctions have important implications for the translation of findings to the human context, necessitating careful interpretation of data derived from mouse studies.

How accurately do findings in human cardiac cell composition and distribution map to the mouse heart? Human heart atlases^9–11^^,57–59^ provide unprecedented insight into the characteristics and functions of myocardial cells in health and disease. Mouse cardiac cell populations have been extensively reviewed since the rise of single-cell technologies.^60–62^ While cardiomyocyte studies require special sample preparation due to their size,^63^ single-cell analysis of the adult mouse heart has focused mainly on fibroblasts, immune, and vascular cells.^14,64–66^ Multiomic approaches are now also highlighting the epigenetic and regulatory differences between the species.^67^

The mouse model is advantageous in the experimental flexibility that it offers to interrogate key questions surrounding the function of myocardial cells. Samples from mice can be obtained from carefully selected time points in development or adulthood. Surgeries can be performed to create disease-like lesions (such as left anterior descending artery ligation to induce MI). Furthermore, genetic engineering can be used to tag marker genes with reporter proteins (for cell-type enrichment by fluorescence-activated cell sorting or lineage tracing) and to engineer gene modifications.

**Figure 4.**
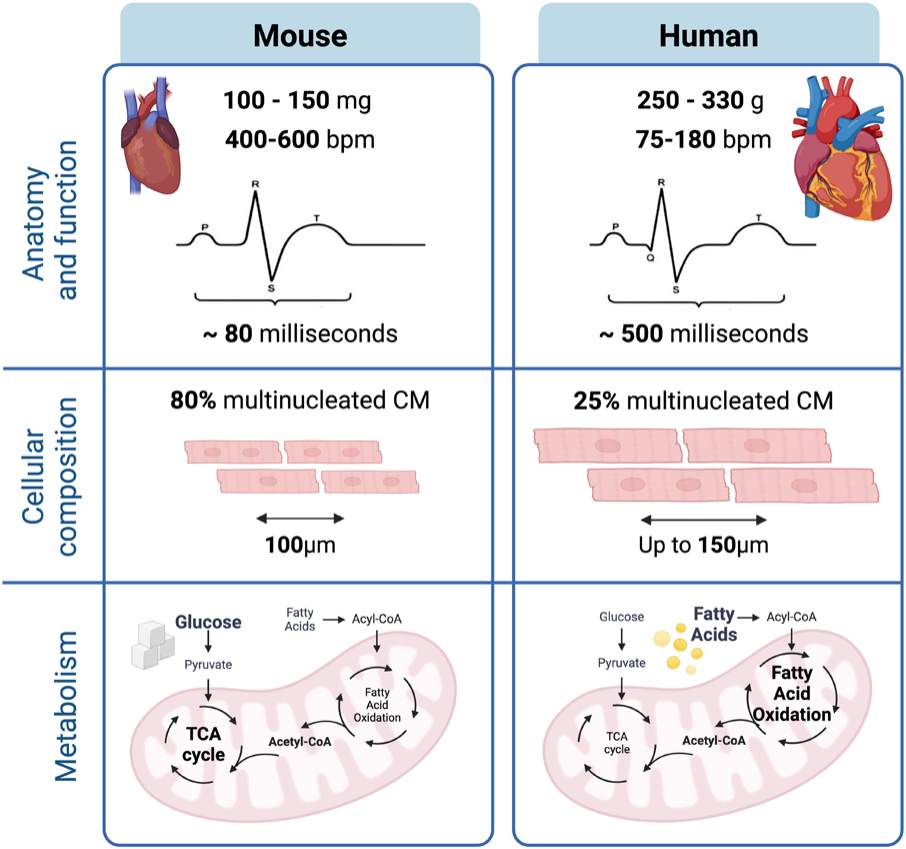
**Major differences between mouse and human hearts, including anatomic, cellular, and metabolic differences.** CM indicates cardiomyocyte; and TCA, tricarboxylic acid.

Studies of the human heart do not have this level of experimental flexibility. Instead, tissue is obtained from deceased or transplant donors, relying on tissue banks or good relationships between research institutions and hospitals. These samples offer rich information in terms of understanding mechanisms and potential treatments for human cardiac diseases. Here, we consider both systems and their implications on single-cell and spatial-omic studies, from anatomic structure to differences in the molecular and genetic levels (Figure 4).

### Anatomical and Mechanical Differences

Although mouse and human hearts share several anatomical similarities and nonmyocyte cell sizes are largely comparable, organ size is a striking difference. Mouse hearts weigh on average between 100 and 150 mg, compared with the human heart, which weighs on average 245 g in women and 331 g in men.^68,69^ Due to the upright position of the human body and the heart resting on the diaphragm, the human heart has a more pyramidal shape. The mouse heart is more ellipsoidal due to its more horizontal position in the pericardial cavity.^70^ The conduction system shows a slight location change, with the sinoatrial node (SAN) located above the right atrium in mice but embedded into the right atrium in human.^71^

The mouse heart also beats at a significantly higher rate, 400 to 600 bpm, compared with the human heartbeat average of 75 to 180 bpm.^70,72^ The difference is partially a reflection of the size as well as other factors, such as metabolic rate. The faster heartbeat is also reflected in the different electrophysiological profiles of mice, with a shorter action potential and differences in the expression of ion channels orchestrating the process. This raises questions on whether mice provide a suitable model for arrhythmias (reviewed in ^73–75^).

### Cellular Composition

The cellular composition of mouse and human hearts and their distinct anatomic locations vary, both in composition and in individual cell states. Initial studies have primarily relied on histology and staining of tissue sections, with a consensus that ≈70% of the myocardial volume is occupied by cardiomyocytes, the contractile muscle cells.^12^ Mouse cardiomyocytes are rectangular cells, 100 µm in length and about 15 to 20 µm in width, while their human counterparts span up to 150 µm in length and contains a more complex T-tubule arrangement system,^76^ allowing for more evenly distributed contraction.

A hallmark of cardiomyocytes is their potential to house multiple chromosome pairs,^77^ increasing their ploidy. This can happen by endoreplication (DNA replication without cell division resulting in increased ploidy), endonucleation (nuclear division without cell division resulting in multinucleated cells), or other processes, such as cell fusion. Adult human cardiomyocytes are primarily mononucleated with 16% to 25% containing more than 1 nucleus, while about 80% of adult mouse cardiomyocytes contain 2 nuclei.^78,79^ In mice, the endonucleation occurs shortly after birth, leading to binucleated cardiomyocytes from a young age with only small increases throughout the lifetime. In humans, polyploidization surges around 10 years of age, with the process continuing throughout life.^77,80^ This variation in nucleation and polyploidy may influence both the proportion and the number of reads per cell in the single-cell studies, as well as well as the chromatin architecture and accessibility landscapes of both bulk and single cardiomyocytes.

Advances in fluorescent cell sorting and more recent single-cell technologies have provided a more detailed overview of the cellular composition of the heart, incorporating many more cell types. However, due to the bias introduced by enzymatic tissue digestion and range in cell sizes, it has proven challenging to reach a consensus on detailed cellular ratios.^81^ While cardiomyocytes are the largest cells in the heart, they account for only 25% to 40% of the cellular fraction.^78,82^ Similarly, the estimations of the fibroblast proportions scale anywhere from 20% to 50% across publications,^12,78,83,84^ marking the endothelial cells as more abundant.^12^

Estimating absolute cellular proportions from scRNA-seq data can also prove challenging. Only a fraction of the original cell suspension is used to generate sequencing data during processing. This is later confounded by quality control, where only cells that survived capture will be considered for compositional analysis, excluding other cells that may be more fragile. Using single nuclei for the estimated proportions removes the size limitation on the cell capture and thus might be better suited for estimations of relative cardiac cell content.

### Metabolic Differences

Mice show a higher cardiac metabolic rate than human hearts, reflected in their higher energetic demand. Mouse hearts depend on glucose metabolism and show higher flexibility in substrate usage, resulting in a different response to metabolic stress and higher resilience to short-term ischemia.^85^ The metabolism of human hearts is more complex by comparison, with the primary source being fatty acids, with the switch to glucose metabolism in perturbations such as heart failure.^86–88^

Different metabolic requirements are particularly prominent in the cardiomyocytes. Murine cardiomyocytes show higher mitochondrial density, which is related to a higher ATP requirement. Their preference for glucose metabolism is reflected in the upregulation of glycolysis and glucose transporters such as *GLUT4* and *HK2*.^89^ In contrast, human adult cardiomyocytes show increased expression of genes involved in fatty acid oxidation, such as *ACOX1*,^90^ and *COX*^91^ and *NDUFA*.^92^

### Genetic Variation

Strain variability in mouse and human genetic diversity significantly impact cardiac research outcomes. Different mouse strains exhibit varied cardiac phenotypes and responses to stress or disease, mirroring the genetic diversity seen in humans.^93^ This variability must be accounted for when extrapolating mouse data to human conditions. Human genetic diversity, influenced by many factors, including ethnicity and environment, presents a challenge in creating universally applicable findings. Thus, acknowledging and integrating this genetic diversity is vital for the accuracy and relevance of cardiac research.

The majority of single-cell studies have focused on the C57BL/6 mouse strain as a commonly accepted background. However, focusing only on one strain can create a bias underlined by the cardiac phenotype specific to this strain. For example, studies have shown that C57BL/6J maintains better cardiac function after ischemia ex vivo while showing fast decompensation in vivo,^94^ while C57BL/6N showed reduced contractile function,^95^ and DBA/2 strain exhibited a more immature cardiac phenotype and was less prone to hypertrophy compared with the other strains.^96^ Striking variation of functional, morphological, and myocardial scar features was also detected across 32 recombinant inbred mouse strains subjected to MI,^93^ which produced marked differences in interstitial cell responses across acute and chronic phases of remodeling postinfarction.^64^ Moreover, analyses of most murine heart failure models are performed in male mice, and whereas heart failure occurs in both sexes to an essentially equal extent and sex-specific differences in cardiovascular remodeling after injury have been documented,^97^ emphasizing the need for a more inclusive experimental design. Comparing cellular features of response to injury across diverse mouse genetic backgrounds thus provides a more realistic model of the variation in human cardiac physiology and disease.

### Cardiac Cells in a Dish: In Vitro Systems

The engineering of cardiac cells and tissue in vitro for applications such as cell therapy and drug screening offers alternative systems to primary cells and animal models.

One of the major avenues is for cardiac cells to be derived by differentiating human-induced pluripotent stem cells (iPSCs) or embryonic stem cells to cardiac lineages using signaling or environmental cues. Examples range from 2-dimensional cultures of cardiomyocytes,^98^ epicardial cells,^99^ and endocardial cells^100^ to self-organizing organoid models with complex 3-dimensional structures.^101^ Clinical implementation of stem cell-based technologies applied to the heart includes the recent development of 3-dimensional cardiac microtissues engineered from iPSCs harboring human mutations, allowing for rapid testing of potential therapeutics for correction of genetic defects and promotion of cardiac regeneration.^102,103^

By guiding cell phenotypes toward desired lineages, these systems provide valuable insight into the cellular mechanisms governing human cardiac cell differentiation and development, and the use of patient-derived iPSCs is useful for understanding pathogenic mechanisms in cells with genetic signatures of disease. However, the ability of these models to faithfully recapitulate in vivo phenotype and behavior is limited; for example, iPSC-derived cardiomyocytes display a fetal-like phenotype that restricts their ability to perform when transplanted in vivo.^104^ While cardiac organoids only partially recapitulate aspects of mature organ architecture and function, they can shed insight into the specification, differentiation, and cellular self-organization during early embryonic development that are less accessible in vivo.^105^

The development of similar systems with mouse stem cells has been less expansive. However, the use of iPSCs from strains with distinct and well characterized genetic backgrounds enables exploration of genetic diversity in in vitro systems.^106^ Furthermore, iPSC traceability to strain enables parallel in vitro and in vivo exploration with a consistent genetic background.^106^

Single-cell omic techniques provide the ability to characterize in vitro systems at a single-cell resolution not attainable with quantitative polymerase chain reaction and bulk assays. Comprehensive atlases of in vivo cardiac cells provide a strong benchmark to evaluate cell types and states present in vitro. However, particularly powerful is the ability to leverage single-cell readouts in pooled perturbation experiments, enabling highly efficient in vitro screening of gene function (eg, Perturb-seq^107^). These have been used in a cardiovascular context to explore functional gene programs in endothelial cells related to arterial diseases.^108^

## CONTEXT: CARDIAC CELLS AND THEIR MICROENVIRONMENTS

**Figure 5.**
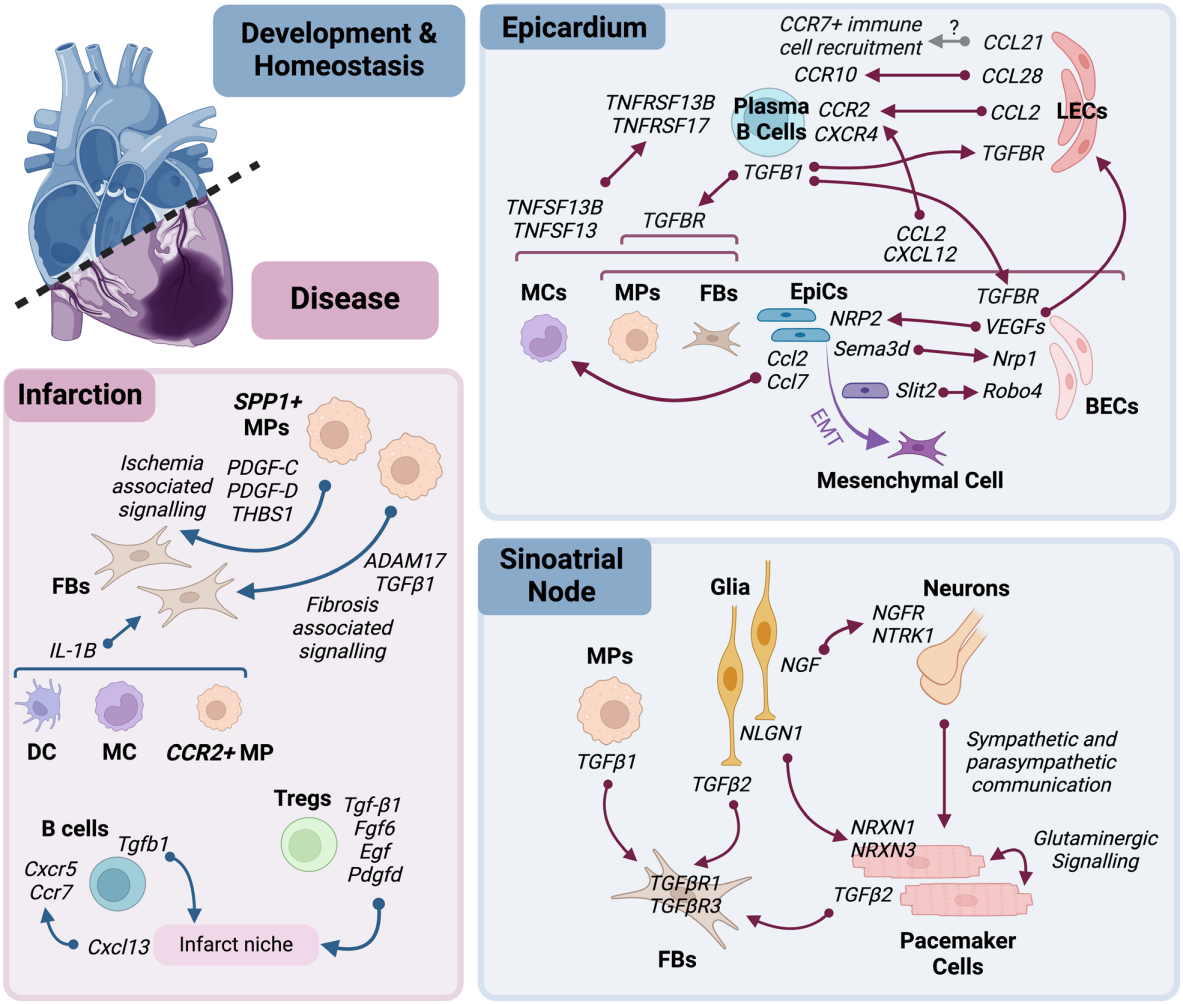
**Coordination of cell types within tissue microenvironments in cardiac tissue in health and disease.** BEC indicates blood endothelial cell; DC, dendritic cell; EMT, epithelial–mesenchymal transition; EpiC, epicardial cell; FB, fibroblast; LEC, lymphatic endothelial cell; MC, monocyte; MP, macrophage; and Treg, regulatory T-cell.

Cells of diverse phenotypes are coordinated to produce niches for highly specialized functions of myocardial tissue. First, we explore the diverse array of cardiac cells in mice and humans and examine their specialization and coordination with other cell types in tissue niches. Subsequently, we consider how single-cell/nuclear and spatial transcriptomic studies have enriched our understanding of these specialized regions (Figure 5).

### Major Cardiac Cell Types in Mouse and Human

The cardiac cellular landscape is characterized by several key cell types contributing to the intricate functioning of the heart. While mouse and human hearts share fundamental cell types, variations exist in their distribution, abundance, and functional characteristics. Detailed studies of adult human subpopulations of these main cell types have been profiled in large-scale atlases.^8,9,57,109^ In contrast, mouse studies are carried out on a smaller scale, and often focus on the developmental stages^110–112^ or specific cell compartments^113^ and anatomical regions^114^ in adults. The major shared cell types and their specific signatures have been recently reviewed by Miranda et al,^115^ and in this section, we focus primarily on the translation of the findings between the species. The differences in resolution between mouse and human studies create a bias in the populations we see across the species and thus should be considered when extrapolating between species.

#### Cardiomyocytes

Cardiomyocytes are the most prominent cell type of cardiac tissue and have been studied for over a hundred years,^116–118^ often in isolation from the nonmyocyte cells. Extensive characterization and comparison of atrial and ventricular cardiomyocytes have been previously reviewed,^119–121^ highlighting their differences in organelle distribution, action potential, and signaling properties. In mouse hearts, single-cell studies have captured predominantly ventricular cardiomyocytes, limiting the observed heterogeneity of this cell type. In contrast, human studies have better addressed this imbalance by sampling atria and ventricles separately.

Large cardiac atlases in humans have shown previously understudied cardiomyocyte heterogeneity in both atria and ventricles, describing several clusters with enriched signatures.^8,9^ Mouse cardiac cell data sets are yet to reach comparable resolutions, initial studies suggest underlying heterogeneity, with the best-described being *Myoz2+* cardiomyocytes with subepicardial localization.^14^ While this population was observed in the fetal human heart,^45,122^ the *MYOZ2* expression in the human heart^9^ data set was dispersed across all cardiomyocyte clusters, signaling a challenge of relying on single markers for translating the similarities across species.

#### Fibroblasts

Fibroblasts are mainly responsible for extracellular matrix (ECM) generation, remodeling, and maintaining the cardiac structural integrity of the cardiac muscle. They are close collaborators of cardiomyocytes and are highly versatile and plastic in their profiles and activation responses.^123^ There are apparent differences between the atrial and ventricular fibroblasts in both mouse and human hearts, with defined differences in activation and remodeling responses.^124,125^ In humans, cardiac fibroblasts have shown remarkable heterogeneity with the number of cell states and activation trajectories, particularly relevant to the disease context.^57–59^

Beyond their interstitial tissue scaffolding functions, fibroblasts regulate organ development, wound healing, and fibrosis and play important immunomodulatory roles in inflammation and self-tolerance.^126^ In mice, an organ-specific set of embryonic cardiogenic transcription factors broadly involved in multiple congenital heart diseases were persistently expressed in isolated adult cardiac fibroblasts, implicating these cells in adult myocardial repair.^127^

##### Interactions of Cardiomyocytes and Fibroblasts

Cardiomyocytes and fibroblasts communicate through an array of signaling molecules. For instance, TGF-β (transforming growth factor-beta), secreted by cardiomyocytes, activates fibroblasts to synthesize critical ECM components during fibrotic responses.^128^ Fibroblasts, in return, secrete collagen and fibronectin, ECM components that significantly influence cardiomyocyte function, including contractility and electrical conduction.^65^ Recent studies also suggest that microRNAs are exchanged between these cell types, modulating gene expression and impacting overall cell function.^129,130^

In mice, multiple cell populations contribute to pathological remodeling of the cardiac ECM.^131^ A single-cell study has implicated cardiomyocytes in fibroblast activation and transition to myofibroblasts.^4^ By comparing ligands with 3-fold greater expression in stressed cardiomyocytes present a day after ischemic injury compared with sham-surgery mice, 15 non-ECM ligands were identified. Looking at receptors among other cardiac cell types, fibroblasts were the most receptive to these ligands. In particular, fibroblasts expressed the highest levels of cognate receptors for *Mfge8*, *Calr*, and *B2m* compared with other cardiac cells. Moreover, fibroblast cultures supplemented with recombinant versions of these ligands increased myofibroblast marker expression. The new roles of the ECM as a signaling and cytokine hub are also emerging, influencing cardiomyocyte behavior and function.^132,133^

#### Endothelial-Perivascular Network

As the most abundant nonmyocyte cell in the heart, endothelial cells line the interior surface of the heart’s blood vessels and are pivotal in regulating vascular functions and cardiac health. In mice, cardiac endothelial cells, as identified by single-cell sequencing, display a gene expression profile geared toward angiogenesis and vascular remodeling. Essential genes like *Cdh5* and *Pecam1* are highly expressed, indicating their active role in maintaining the microvascular network and adapting to changes in metabolic demand.^65^ Conversely, human cardiac endothelial cells exhibit a more diverse range of subtypes, each specialized for particular functions within different vascular segments.^9^ This diversity is crucial for supporting the human heart’s demand for oxygen and nutrients, with genes related to endothelial barrier function (such as *CDH5*) and leukocyte trafficking (such as *SELE*) being prominently expressed. Notably, endothelial cell profiles are heavily influenced by aging, during which angiogenic capacity is lost, which may confound results when the relatively young age of analysis in mouse models is not taken into account. Nevertheless, these differences highlight endothelial cell species-specific roles in coronary artery function, capillary exchange, and response to ischemic conditions.

Perivascular cells, including smooth muscle cells and pericytes, are integral to the heart’s vascular structure. In mouse hearts, perivascular cells regulate microvascular stability and blood flow. Single-cell analyses reveal a high expression of contractile and adhesion proteins such as *ACTA2* in smooth muscle cells and *VNT* in pericytes,^65^ underscoring their role in vascular tone regulation. These cells also show angiogenesis and remodeling involvement, especially in response to cardiac tissue-changing metabolic and oxygen needs.^134^ In human hearts, perivascular cells demonstrate a more complex gene expression profile, indicative of their multifaceted roles in maintaining vascular stability and intercellular communication within cardiac tissue. Genes regulating ECM components like elastin or contraction like calponin,^9^ emphasize their contribution to vascular and cardiac tissue integrity. This complexity suggests their significant involvement in pathologies such as hypertension and atherosclerosis, where altered perivascular cell function contributes to disease progression.^135^

#### Immune Cardiac Compartment

Both mouse and human hearts are equipped with immune cells that contribute to cardiac homeostasis and response to injury. However, the immune cell composition, activation patterns, and inflammatory responses may vary between species. Recognizing these distinctions is critical for comprehending immune-mediated processes in the context of cardiac diseases and therapeutic interventions, and single-cell analysis has significantly enhanced the understanding of these cells in the cardiac context, providing insights into their roles in health and disease.

Myeloid cells, including macrophages, dendritic cells, and granulocytes, form a significant part of the cardiac immune landscape and are key players in the inflammatory response to acute injury. Cardiac macrophages, identified by markers like *CD68* in humans and *F4/80* in mice, are key players in tissue homeostasis, injury response, and repair. They exhibit remarkable plasticity and can adopt proinflammatory or anti-inflammatory phenotypes depending on the cardiac microenvironment.^136^ scRNA-seq has been pivotal in categorizing these myeloid cells into distinct subsets, each with specific roles in cardiac physiology and pathology, and revealing their dynamic changes in atherosclerosis^137^ and different cardiomyopathies.^57–59,138^

Lymphoid cells play crucial roles in the cardiac environment. Single-cell technologies have revealed the diversity of these lymphoid populations in mice, including various subsets with distinct functional roles.^139^ Using the same technologies in human hearts, lymphoid cells exhibit a similar diversity, but with highlighted differences in their distribution and functional responses. T cells, particularly regulatory T cells and γδ T cells, in human hearts are involved in immune surveillance and modulating inflammation and fibrosis, particularly in conditions like myocarditis.^140^ Recent studies have highlighted the importance of previously overlooked populations, such as cardiac B and plasma cells^141^ which are believed to contribute to the pathogenesis of certain autoimmune and inflammatory heart diseases. Although the role of innate leukocyte cells and other innate immune cells remains to be elucidated, they have gained attention for their role in the immune response to MI^142^

The interactions between immune and cardiac cells (such as cardiomyocytes, fibroblasts, and endothelial cells) are complex and critical for the heart’s response to stress and injury. For example, activated macrophages can secrete cytokines and growth factors that influence cardiomyocyte function and fibroblast activation, leading to tissue remodeling or fibrosis.^143^ Similarly, interactions between T cells and endothelial cells can play a role in vascular inflammation and atherogenesis.^144^ While the early immune response to myocardial injury is essential to maintain tissue integrity and to avoid fatal cardiac rupture, the longer-term action of dendritic cells, responding to cardiomyocyte necrosis, presents cardiac antigen to T cells and initiates anti-cardiac autoreactivity of the adaptive immune system that can exacerbate structural remodeling, functional decline, and heart failure.^145^ This interplay of innate and adaptive immune cell responses to cardiac injury and disease is a ripe target for novel interventions to prevent postischemic immunopathology.^146,147^ Single-cell studies have shed light on these critical cellular interactions, revealing how alterations in immune cell behavior can contribute to various cardiac pathologies and suggesting potential therapeutic targets to modulate these interactions for treating heart diseases.

In conclusion, an in-depth exploration of cardiac cell types in mice and humans reveals shared fundamental components alongside species-specific characteristics. Acknowledging these nuances is essential for enhancing the translational relevance of experimental findings from mouse models to human cardiac biology.

### Cardiac Tissue Microenvironments

Single-cell and spatial transcriptomic approaches offer the opportunity to move beyond the study of isolated cell types toward an understanding of tissue microenvironments. This requires the piecing together of coordinated cell types, the signaling pathways that connect them, and the matrix and environment in which they interact. Cell:cell communication inferred from single-cell data sets can be contextualized with knowledge of distribution of cell types and cell neighborhoods from spatial approaches.^148^ Figure 5 summarizes key signaling interactions in three cardiac tissue niches explored using single-cell and spatial approaches.

#### SAN in Homeostasis

The SAN, the pacemaker of the heart, establishes sinus rhythm that is propagated across the atria and toward the atrioventricular node, ensuring coordinated contraction of the atria and, subsequently, the ventricles.^149^ It appears as a distinct fibrotic structure in the right atrium of both humans^149^ and mice.^150^ The pathogenic role of the SAN has been linked to several arrhythmia disorders,^149^ making it an important functional and therapeutic target.

It is well established that the fibrotic component contributes approximately half of the SAN structure, and the cardiac pacemaker cells, specialized cardiomyocytes, are the origin of pacemaker activity.^149^ However, there is great cellular heterogeneity within the structure. Single-cell and spatial transcriptomic studies have been applied to resolve the cell populations of the SAN in both mice and humans (Table). The scarcity of SAN cells poses technical challenges; targeted approaches, such as extensive histological stainings and microdissection of the SAN region, were necessary to collect sufficient cell numbers in both mouse^151,152^ and human^10^ studies.

**Table.**
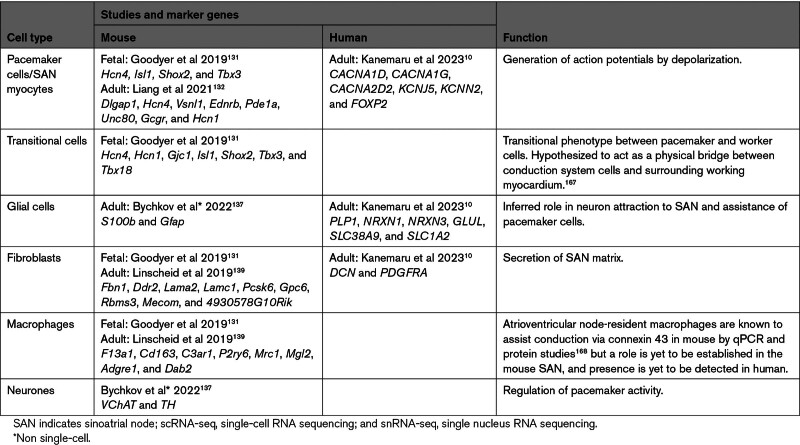
SAN Cell Types Defined by scRNA-seq, snRNA-seq, Multiome, or Immunofluorescence Studies

In SAN, diverse cell types interact to form interconnected networks that contribute to function. Below, we explore specific cell:cell interactions and their functional implications.

##### Glia, Pacemakers, and Neurons

Pacemaker cells are specialized cardiomyocytes with automaticity that enables spontaneous depolarization.^153^ As the origin of the SAN’s electrical activity, these cells have been the key focus of single-cell studies of the SAN (Table). However, the mechanism of how pacemaker cells bring about SAN depolarization is highly speculated (reviewed in Lakatta et al^154^).

Several studies have focused on how pacemaker cells interact with different cell types in the SAN to influence depolarization, such as fibroblasts.^155^ Additionally, the SAN receives neural input from the dendrites of neurones that have their bodies in the right atrial ganglionated plexus.^156^ Manually annotated Visium spots of the right atrial ganglionated plexus with high levels of neuronal cytoskeletal markers (indicative of neuron content) were correlated with the expression of both cholinergic (parasympathetic, *SLC18A3*, *SLC5A7*) and catecholaminergic (sympathetic, *TH*, *DDC*) markers.^10^ Pacemaker cells were found to express the corresponding receptors for both the cholinergic and catecholaminergic markers.^10^ A recent immunostaining study in mice detected cholinergic and adrenergic neurones within the SAN forming a network around *HCN4*+ pacemaker cells,^157^ in line with the findings in human SAN.

The role of glial cells as an organizer of pacemaker and neural interactions is beginning to be elucidated. A cross-species study of cardiac astroglia suggested that these cells contribute to heart development, innervation, and regulation of heart rate.^158^ Skelly et al^65^ were the first to resolve a glial population in the mouse heart at the single-cell level. In humans, a cardiac glial population was found to express *NGF*, required for neuronal function, and its receptors *NGFR* and *NTRK1* were expressed in the right atrial ganglionated plexus.^10^ This indicates that neurons with the potential to span from the right atrial ganglionated plexus to the SAN express receptors for a key neural factor secreted by glia, suggesting a role for glia in secreting factors that maintain innervation in the SAN.

Glial cells also interact with pacemaker cells. A recent immunohistochemical study in mice captured Gfap+ Sb100+ double-positive glia forming into a web-like structure around *Hcn4+* pacemaker cells of the SAN.^157^ In humans, glial cells have been primarily characterized by *PLP1* expression, and cells expressing this marker have been found in the SAN, colocating with *HCN1*+ pacemaker cells.^10^ This colocalization has been supported by the IHC staining, hinting at communication between the 2 cell types. Cell-cell interactome analysis predicted neurexin-mediated interaction between *NRXN1/3+* glial cells and pacemaker cells expressing ligand *NLGN1*.^10^

These single-cell and spatial studies shed light on the pivotal role of glial cells in maintaining neural connections to the SAN in mice and humans, closely interacting with pacemakers, and assisting in the local signaling pathways.

##### SAN Cell Interactions With the Matrix

The SAN has a large ECM component with approximately half of the SAN in humans being fibrotic.^149^ A recent proteomic study of the mouse SAN describes the matrix as highly elastic and rich in fibronectin and collagen IV.^159^ The matrix has an important role in maintaining pacemaker phenotype: human-induced pluripotent stem cell–derived cardiomyocytes transplanted into an acellular porcine SAN matrix were found to improve their pacemaker phenotype.^160^

Studies at the single-cell level provide the opportunity to understand the cellular and molecular contributions to the matrix, which is primarily secreted by the SAN-based fibroblasts (Table). Activated fibroblasts have previously been primarily related to pathological conditions and cardiac fibrosis,^57,59^ but in the SAN, they provide an ECM production hub necessary for the electrical isolation. *NGF*+ glial cells, pacemaker cells and a specific type of macrophage (*LYVE1+IGF+*) have all been found to produce signaling molecules and ligands targeting these fibroblasts, particularly through the *TGF-β* signaling, which regulates many ECM synthesis genes (such as *COL1A1* and *FN1*).^10^ *TGF-β* binding proteins have also been implicated in cooperation with fibrillin, collagen VI, and elastin to confer elastic properties on the matrix by electron microscopy,^159^ highlighting the importance of this central signaling pathway in the SAN niche.

#### Epicardial Niche as a Signaling Hub

The epicardium is the outer mesothelial layer surrounding the myocardium. It is derived from cells of the proepicardial organ that migrate and surround the heart.^161^ The epicardium actively contributes to myocardial tissue in three key contexts. First, the epicardial cells undergo epithelial-to-mesenchymal transition (EMT) to give rise to the development of myocardial cell types, including smooth muscle cells, fibroblasts, and, speculatively, cardiomyocytes.^162^ Epicardial signaling and epicardial-derived cells also contribute to vascularization.^37^ Second, the epicardium is a paracrine signaling hub with a homeostatic immune barrier role.^10,163^ Lastly, the epicardium plays an important role in the pathological conditions, known for its regenerative properties in zebrafish, which is not the case in human.^164^

The mesothelial cells of the epicardial layer are varied in properties and phenotypes, which are often context-dependent. In development, it has long been established that epicardial cells express *WT1* and *TBX18*, important for EMT and vascularization of the heart, respectively.^162^ These markers, among others, have been used to identify epicardial clusters in human^163,165^ and mouse^37,110^ single-cell heart studies. New markers, such as *NPY*, have been described in mice.^165^ However, single-cell studies of both species have unveiled previously unexplored diversity of epicardial cell transitional states in the human and mouse heart, particularly in development and disease. For example, single-cell studies of the human epicardium suggest that separate subpopulations of fibroblast-like and proliferative epicardial cells are present in fetal epicardium and absent in adult epicardium.^163^ In the mouse infarct heart, 11 separate epicardial subpopulations were detected, including those with an immune response-modulating and fibroblast-like phenotype.^166^

The epicardium is an important paracrine signaling hub in development, homeostasis, and disease.^162^ Cell-cell interactome analysis of a human epicardial data set showed a significantly higher number in fetal compared with adult cells, indicating a crucial signaling role in development.^163^ These interactions include a complex network of cellular signaling between epicardial cells and endocardial cells, endothelial cells, and neurons with collagens as a major signaling molecule.^163^ Below, we explore examples of how epicardial paracrine signaling assists myocardial tissue function.

##### Epicardial Communication

The epicardium physically isolates myocardial tissue from the external environment, and its resident immune population acts as a biological defense against external pathogens and is involved in wound healing post-MI.^167^ A microenvironment of epicardial cells, macrophages, endothelial cells, and fibroblasts is predicted to cooperate in modulating this immune response.

The secretion of immune-attracting chemokines by epicardial cells has been identified in single-cell studies of the adult human and mouse infarct hearts. In human epicardial cells, an age-associated gene module enriched for genes associated with the immune response, was identified to be more highly expressed in adults compared with fetal epicardium.^163^ In mouse postmyocardial infarct hearts, an epicardial population characterized by interferon response showed *Ccl2* and *Ccl7* expression, which are involved in monocyte recruitment.^166^

Using a spatial transcriptomic approach, Kanemaru et al^10^ mapped plasma B-cell populations identified in snRNA-seq data to sections of the epicardial-subepicardial structure and predicted interactions of plasma B cells with other cells in the epicardial niche. Using spatially resolved CellPhoneDB analysis,^168^ an interaction was predicted between plasma B cells that express receptors *CCR2* and *CXCR4* with ligands *CCL2* and *CXCL12*, respectively, secreted by endothelial cells, fibroblasts, mesothelial cells, and macrophages.^10^ The protein products of *TNFSF13B* (BAFF) and *TNFSF13* (APRIL) expressed in macrophages, monocytes, and fibroblasts are predicted to interact with respective receptor counterparts *TNFRSF13B* and *TNFRSF17* expressed in plasma B cells.^10^ Indeed, single molecule fluorescence in situ hybridization showed plasma B cells to express *TNFRSF13B* and macrophages co-locate in the epicardium.^10^ Further interactions were predicted: lymphatic endothelial cells express *CCL28*, which may serve as a recruitment mechanism for IgA+ *CCR10*-expressing plasma B cells; fibroblasts, subsets of macrophages, and endothelial cells express receptors for *TGFB1*, which is secreted by plasma B cells (Figure 5).^10^

The epicardium contributes to vascularization by producing key cellular components (endothelial and SMCs and pericytes) and secreting paracrine signals that stimulate vessel development and specification.^169^ Trajectory analysis of mouse epicardial cells in development showed a progenitor epicardial pool following two trajectories: EMT to become mesenchymal cells or maturation to a surface-resident mesothelial population.^37^ Subpopulations of epicardial and epicardial-derived cells along this trajectory expressed distinct angiogenesis-regulating factors.^37^ *Sema3d* was found to be enriched in the mesothelial populations of epicardial-derived cells. Expression of its ligand, *Nrp1*, is present in all endothelial cell clusters, although *Sema3d* expression is restricted to the epicardial surface. This indicates a putative epicardial surface epicardial:endothelial cell interaction via *Sema3d* and *Nrp1*. The expression of another ligand, *Slit2*, which was previously found to stimulate vasculature formation, becomes enriched along the EMT trajectory. *Slit2* expression has been localized and is also present in a population of cells in close contact with endothelial cells that express receptors for the *Slit2* ligand, *Robo4*, by single molecule fluorescence in situ hybridization. *Sema3d* has previously been implicated in cardiac angiogenesis and pulmonary vein patterning,^170^ and *Slit2* has been implicated in vascularization in other contexts.^171^ The study by Quijada et al enabled the secretion of these ligands from the epicardium to be studied in an EMT-stage–resolved manner. In human fetal epicardium, an interaction was predicted between *NRP2* expressed by epicardial cells and vascular endothelial growth factors (VEGFs) secreted by endothelial cells.^163^

The cardiac lymphatic system, responsible for maintaining fluid and immune cell homeostasis within the heart, has vessels spanning from the subepicardial to subendocardial spaces.^172^ Recent single-cell studies of the developing human heart suggest an emerging landscape of communication involving the cells of the monolayer wall of lymphatic vessels, lymphatic endothelial cells, in the subepicardial niche. To profile the development of the lymphatic vessels alongside the coronary artery vasculature, a recent study performed multiomic profiling (snRNA/ATAC-seq) of subepicardial endothelial cells in fetal hearts at 10 to 11 postconception weeks.^173^ This study proposed an interaction between *VEGF*+ arterial endothelial cells and lymphatic endothelial cells that shows a gene ontology analysis term enrichment for *VEGF* signaling; arterial endothelial cells may guide lymphatic endothelial cell differentiation to develop the lymphatics surrounding the coronary arteries.^173^ Related to the immune function of lymphatic vessels, *CCL21*+ lymphatic endothelial cells have been detected by a single-cell study of endothelial cells in ventricular sections.^174^ Staining of human epicardial sections has indicated *CCL21* expression in the left ventricle epicardial region.^10^ Previous studies have indicated communication between *CCL21*+ lymphatic endothelial cells and *CCR7*+ immune cells, including dendritic cells^175^ and lymphocytes.^176^ This hints at an additional immune recruitment mechanism in the subepicardial niche.

#### Cardiac Niches in the Infarct Heart

Cardiac cells and tissue undergo dramatic changes following MI and other cardiac disorders.^115^ To facilitate processes such as immune activation, fibrotic scar formation, and vascularization, molecular niches are altered. At the cellular level, single-cell and spatial transcriptomic studies of the infarct heart identify both homeostatic and disease-activated populations of the same cell type for a range of cardiac cells, including cardiomyocytes, fibroblasts, endothelial cells, and immune cells in both mouse^4,14,64,66,177–179^ and human.^11,180^

At the niche level, Kuppe et al^11^ provide the most rigorous investigation of the molecular niches in MI to date. From 31 heart samples, infarct and healthy, nuclei were extracted, and cryosections were taken for spatial transcriptomics. To identify cell-type niches across the samples, Visium spots from the spatial transcriptomic sections were clustered based on cell type composition and gene expression, identifying myogenic, inflammatory, and fibrotic niches.^11^

To obtain further molecular detail, the signaling processes within niches could be predicted by studying colocalized signaling pathways, or signaling pathways spanning between neighboring niches, identifying key fibrotic (TGF-β and NFκB) and immune (JAK-STAT processes).^11^ Below, we explore how single-cell and spatial transcriptomics can elucidate disease processes’ molecular details.

##### Fibroblast Activation in the Infarct Heart

As discussed, single-cell studies of MI reveal both diseased and healthy populations of many cell types. The application of spatial transcriptomic analysis to samples from human ischemic hearts has identified fibroblasts differentially colocalising with smooth muscle cells, highlighting the interactions diferences in health and disease.^11^

The enrichment of myeloid clusters, as revealed by spatial analysis^11^ is another key characteristic of the fibrotic niche in ischemic cardiac tissue, including clusters expressing *SPP1* that can activate fibroblasts in vitro. In the cardiac ischemic context, *SPP1*+ macrophages are better predictors of fibroblast state than other myeloid cells: gradients of myofibroblast markers aligned with those expressing *SPP1*, which lie adjacent to myofibroblasts.^11^ The same study also uncovered alterations in predicted cell:cell communication between *SPP1*+ macrophages and fibroblasts in disease (fibrotic/ischemic) states compared with myogenic samples from unaffected areas, as indicated by the upregulation of *PDGF-C*, *PDGF-D*, and *THBS1* signaling in ischemic compared with myogenic samples, whereas *ADAM17* and *TGFB1* were upregulated in fibrotic compared with myogenic samples. Although the predicted roles for these signaling pathways in fibroblast activation require further exploration, macrophage signaling to activated fibroblasts has been linked via single-cell studies in other disease contexts, such as cardiomyopathy and hypertrophic cardiomyopathy,^10^ and reframing these observations in a spatial context is an important step in classifying clinically relevant cell niches to understand organ function.

Further multiomic studies have explored immune IL-1β–mediated fibroblast activation. In humans, cell:cell communication analysis in scRNA-seq data was used to predict IL-1β signaling from *CCR2+* monocytes, macrophages, and classic dendritic cells to activated fibroblasts.^181^ Spatial transcriptomics was used to identify an immune-fibro niche containing the aforementioned cell types, enriched in Nf-κB, a downstream signal of IL-1β.^181^ Another study in mouse leveraging snATAC-seq (single-cell transposase-accessible chromatin with sequencing) mechanistically investigated this activation and suggested that IL-1β secreted by *Cx3cr1* positive-myeloid cells affects transcription factor binding at an enhancer proximal to *Meox1* locus, which is crucial in the fibrotic response.^182^

##### Lymphocyte Communication in the MI Niche

Recent multimodal studies integrating scRNA-seq with receptor sequencing have enabled deep profiling of lymphocytes within the mouse infarct niche.

CD4+ T cells with T-cell receptors against myosin heavy chain α (T-cell receptor M cells) have been shown to differentiate into regulatory T cells (Tregs) with cardioprotective effects in the mouse infarct heart.^183^ Combined scRNA-seq and T-cell receptor sequencing profiling of Tregs derived from adoptively transferred T-cell receptor M cells suggested infarct niche-specific differentiation to either a profibrotic phenotype (expressing *Tgfb1*) or immune checkpoint inhibition phenotype (with expression of *Pdcd1*, *Ctla4*, and *Tigit*), and a similar pattern was also observed in endogenously derived Tregs.^184^ Subsequent bulk RNA-seq and flow cytometry hinted at Treg communication with fibroblasts and endothelial cells (via *Fgf6*, *Egf*, and *Pdgfd*) and lowered the expression of proinflammatory cytokines.^184^ Treg function has also been explored in the mouse poststroke brain, demonstrating Treg function in another ischemic niche.^185^

A similar investigation of B cells in the mouse heart 5 days post-MI using scRNA-seq and B-cell receptor-seq revealed polyclonality among B cells, suggesting a lack of myocardial-driven specificity and expansion.^186^ However, scRNA-seq profiling resolved a heart-specific B-cell population (hB cells) located in myocardial scar tissue, with expression of *Tgfb1* and receptors *Cxcr5* and *Ccr7*, which peaks in cell numbers at day 7 post-MI but is not present in nonmyocardial tissue.^186^ Bulk methods were used to confirm the presence of ligands to *Cxcr5* and *Ccr7* in scar tissue, including *Cxcl13*, where anti-CXCL13 antibody administered to mice reduced B-cell localization to the heart post-MI.^186^ This hints at a *Cxcl13*/*Cxcr5* axis-mediated recruitment of B cells to the heart.

More studies are needed to investigate the in-depth communication of lymphocytes within the MI niche in situ. Given the small size of these cells, the era of probe-based methods that resolve transcripts at the single-cell and single molecule level will pave the way to exploring interactions in this microenvironment.

## FUTURE PERSPECTIVE AND DISCUSSION

As we advance our understanding of cardiac biology through single-cell and spatial omics, several important areas emerge for future exploration. The future of cardiac cellular niches lies in unraveling their dynamics at even higher single-cell resolution, as ongoing studies reveal new cell types, focus on new regions, and use more diverse models and populations, expanding our understanding of how these cells and their interactions evolve during development, disease progression, and in response to therapies. Advances in spatial studies go hand in hand with continuously placing cells into their environments and niches and unraveling novel complexities and interconnectivities.

### Unifying Cell Types Across Species

While established markers provide a strong foundation for identifying major cardiac cell types, the ability of single-cell technologies to resolve a spectrum of cellular heterogeneity within these populations can present several challenges. For example, the nomenclature used to describe these subpopulations may vary across studies and species, preventing ease of identification of previously defined subpopulations in newly generated data sets. To address this challenge, a concerted effort is required to continuously define robust consensus markers for emerging cell populations and subpopulations/states and to develop a harmonized cellular taxonomy for the heart. An integrative heart atlas effort, incorporating a significantly greater number of cells from healthy and diseased hearts across different species, will significantly advance this goal. Such a large-scale reference would provide a common language for the field, enabling researchers to compare findings more effectively and accelerate our understanding of cardiac cell biology in health and disease.

### Systemic Perspective and Intertissue Niche Communication

With the rise of the atlases of whole organisms, such as Tabula Muris^187^ and Tabula Sapiens,^188^ we are starting to adopt a more holistic perspective, considering the heart not in isolation but as part of an integrated organ network. The interactions between different organs, such as the brain-heart axis^189^ or the interplay between cardiac function, microbiome, and renal physiology,^190^ are areas ripe for exploration. Understanding intertissue niche communication, encompassing endocrinology and systemic metabolism, will be the goal of the next few years, where larger cohorts and more targeted sampling will bring us closer to the sex differences and their implications for more personalized medicine. These systemic perspectives can unravel how global physiological changes influence cardiac niches and vice versa.

### Need for New Models

Mice and other animal models have long been used to elucidate the mechanisms of cardiac health disease and have provided essential insights that would not have been possible to study in humans. While underlying species-specific differences should always be considered when extrapolating those findings, these can be used as an advantage, providing a new perspective rather than a limitation. A growing appreciation for the profound influence of genetic variation on human disease risk underscores the need to reach beyond current mouse models largely built on the standard C57BL/6 strain to better represent the nuanced differences cardiac biology often presents,^93^ and to reflect the genetic and environmental complexity of human pathophysiological processes.

There is always room for improvement, however, as most mouse models of cardiovascular disease are evaluated at a much younger age than the predominantly advanced age of most terminal heart failure patients. Just as differences in the timing of the fatty acid versus glycolytic metabolism switch in fetal human versus mouse hearts can confound comparative analyses, different outcomes between species may be influenced by a lack of major comorbidities in young mice that often accompany human cardiovascular disease (eg, aging, insulin resistance, hypercholesterolemia, and hypertension). Thus, more well-matched cross-species comparisons that take these features into consideration may yield more informative results.

Beyond animal models, in vitro cellular and organoid microengineered models that mimic these dynamic niches will be instrumental in dissecting the cellular crosstalk and metabolic exchanges within the heart.^191^ The development of new in vitro models informed by single-cell studies is essential for advancing cardiac research. These models can also help in understanding how external stimuli, like pharmacological agents, influence cardiac cell behavior and niche dynamics. By mimicking the complexity of the cardiac environment, through encompassing several cardiac cell types, we can study cellular interactions, responses to stress, and the testing of therapeutic interventions.

### Emerging Technologies and Data Analysis

Cardiac research in the post-genome-wide association study era has seen a shift in focus from cataloguing genetic variation to multiomic studies of gene function that are strengthened by comparative analysis of humans and genetically diverse mouse panels to study natural genetic and phenotypic variation in a controlled environment.^192,193^ The field is set to benefit immensely from advancements in technologies such as high-definition spatial transcriptomics^115^ with a goal to improve resolution of gene expression down to the single-cell level. New and improved modalities alongside the transcriptome, such as genome regulation, will provide deeper insights into the epigenetic landscape, spatial organization and real-time dynamic gene expression in cardiac tissues. High-throughput proteomic approaches^194^ have sharpened our understanding of cardiac gene function, as similar patterns of gene expression between tissues do not always predict similar function.^195^ Indeed, differences in wiring between RNA and protein coexpression networks have revealed that functionally coherent RNA modules among tissues are more tightly linked by protein coexpression,^196,197^ suggesting that multiomic approaches may ultimately provide a more accurate view of functional relationships in cells of the heart.

Integrated with machine learning and artificial intelligence algorithms, clinical imaging techniques offer the potential to study cardiac tissue architecture and function in vivo noninvasively. Advanced data analysis techniques and the creation of comprehensive models, such as digital twins, will revolutionize the modeling of cardiac pathways and disease progression. Integrating these research findings with clinical perspectives is essential for translating laboratory discoveries into therapeutic interventions.

The future of cardiac research, propelled by single-cell and spatial omics, offers exciting possibilities. By delving deeper into cellular niches, adopting a systemic perspective, developing new in vitro models, embracing emerging technologies, and integrating developmental and clinical insights, we are poised to unravel the complexities of the heart like never before. This integrated approach will enhance our understanding of cardiac biology and pave the way for novel diagnostic tools and therapeutic strategies, ultimately improving patient care and outcomes in cardiac diseases.

## ARTICLE INFORMATION

### Acknowledgments

The authors thank Dr Carlos Talavera-López, Dr Kazumasa Kanemaru, and Dr James Cranley for proofreading the document and for the many valuable comments and suggestions. Illustrations were created with Biorender, and further edited by Dr Clara Borràs Eroles.

### Sources of Funding

J.A. Palmer is funded by the British Heart Foundation (4-year PhD Studentship) and the Wellcome Trust. N. Rosenthal is funded by the Leducq Foundation. M. Litvinukova is funded by CRC1525 by Deutsche Forschungsgemeinschaft (DGF) (453989101). S.A. Teichmann is funded by the Wellcome Trust (WT206194).

### Disclosures

S.A. Teichmann has consulted for or been a member of scientific advisory boards at Qiagen, Sanofi, GlaxoSmithKline, ForeSite Labs, Genentech, Biogen, and Roche. She is a consultant and equity holder for TransitionBio and EnsoCell. She is employed part-time by GlaxoSmithKline. The other authors report no conflicts.
